# Multilayer Proteome and Metabolome‐Based Validation Uncovers Combined Regulatory Roles and Predictive Values of 6 RNA Modifications and Cellular Senescence in Alzheimer's Disease

**DOI:** 10.1002/cns.71021

**Published:** 2026-07-14

**Authors:** Mengjie Tian, Ke Ye, Xinyi Chen, Lulu Liu, Xinyu Han, Tianhu Zheng, Xu Gao, Qing Xia, Fuyuan Li, Dayong Wang

**Affiliations:** ^1^ Department of Biochemistry and Molecular Biology, School of Basic Medical Sciences Harbin Medical University Harbin Heilongjiang China; ^2^ Department of Pulmonary and Critical Care Medicine, Institute of Respiratory Health, Frontiers Science Center for Disease‐Related Molecular Network, State Key Laboratory of Respiratory Health and Multimorbidity West China Hospital, Sichuan University Chengdu Sichuan China; ^3^ Key Laboratory of Heilongjiang Province for Genetically Modified Animals Harbin Medical University Harbin Heilongjiang China; ^4^ Dongzhimen Hospital, Beijing University of Chinese Medicine Beijing China; ^5^ Center for Endemic Disease Control, Chinese Center for Disease Control and Prevention Harbin Medical University Harbin Heilongjiang China; ^6^ NHC Key Laboratory of Etiology and Epidemiology (Harbin Medical University) Harbin Heilongjiang China; ^7^ Joint Key Laboratory of Endemic Diseases (Harbin Medical University, Guizhou Medical University Xi' an Jiaotong University) Harbin Heilongjiang China

**Keywords:** Alzheimer's disease, cellular senescence, machine learning, metabolism, multi‐omics analysis, RNA modification

## Abstract

**Aims:**

Existing studies have revealed that RNA modification regulators and cellular senescence can affect the Alzheimer's disease (AD) process. This study investigated the synergistic mechanism in the brains of AD.

**Methods:**

Based on brain tissue proteomics of patients with AD, we screened out the subtypes of patients that are coordinately regulated by cellular senescence‐related proteins and RNA modification regulators. Transcriptome datasets were used to validate and evaluate 20 hub proteins identified using 100 integrated machine learning algorithms. Finally, protein and metabolic data were employed to explore the characteristics of metabolic subtypes and pathways in AD progression.

**Results:**

The diagnostic model had good diagnostic performance, as revealed by the average area under the receiver operating characteristic curve (AUC) = 0.885 of the internal datasets and the average AUC = 0.89 of transcriptome datasets. Risk score can be used to assess disease progression and the corresponding changes in metabolic characteristics. Finally, metabolic analysis indicates significant abnormalities in amino acid and lipid metabolism during the progression of AD.

**Conclusion:**

We revealed the potential role of RNA modification regulators and cellular senescence‐related proteins in AD pathogenesis and related diagnostic markers through proteomic analysis and machine learning‐based methods.

## Graphical abstract

This study integrated multi‐omics data to analyze the synergistic effects of cellular senescence and RNA modifications on AD pathology. Transcriptome and proteome data were used to validate the stability of machine learning‐screened proteins and the model, while metabolomics data further revealed metabolic alterations during the progression of AD pathology.

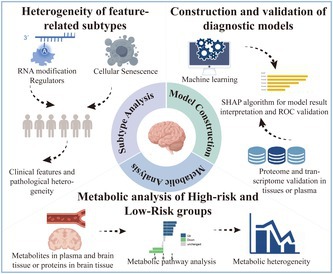

**Abbreviations**
AD: Alzheimer's disease
BBB: Blood–brain barrier
CDR: Clinical dementia rating
GO: Gene ontology
GSEA: Gene set enrichment analysis
GSVA: Gene set variation analysis
KEGG: Kyoto encyclopedia of genes and genomes
m1A: N1‐methyladenosine
m5C: 5‐methylcytosine
m5U: 5‐methyluracil
m6A: N6‐methyladenosine
m6Am: N6, 2′‐O‐dimethyladenosine
m7G: N7‐methylguanosine
WGCNA: Weighted correlation network analysis
WP: Wiki pathways

## 1 Introduction

AD is a type of neurodegenerative disorder. The prevalence of AD gradually increases with age, and its main clinical manifestation is dementia [1, 2]. The studies have depicted that cellular senescence and RNA modifications are commonly observed in various neurodegenerative diseases [3, 4, 5, 6]. Consequently, the study of cellular senescence and RNA modification in the brain of patients with AD deserves attention.

Recently, chemical modification of RNA has been considered a vital mechanism for regulating gene expression and protein translation. Multiple RNA modifications, including m1A, m5C, m5U, m6A, m6Am, and m7G, have recently attracted increasing attention [7]. Therefore, research into their combined effects on neurodegenerative diseases is necessary. This project mainly studied the joint effects of m6A, m1A, m5C, m5U, m6Am, and m7G, six RNA‐modified proteins, on AD pathology and cognition.

Cellular senescence, a hallmark of aging, is a major risk factor for AD [8, 9]. Transcriptome analyses of AD patients and aged brains have shown that neurons and other CNS nonneuronal cells exhibit senescence‐associated phenotypes [10, 11]. Additionally, studies on abnormal lipid metabolism in glial cells offer new insights into AD pathogenesis [12]. Thus, investigating metabolic changes during cellular senescence and disease progression can deepen our understanding of AD pathogenesis [13, 14]. Despite observations of aberrant RNA modification profiles and senescent cell accumulation in brain tissues, their underlying molecular mechanisms remain unclear.

To address this challenge, we evaluated the combined effects of six RNA modifications and cellular senescence in AD. We developed a diagnostic model for patients with AD named CSRMP based on cellular senescence‐related and RNA modification regulatory proteins, emphasizing the role of these two proteins in the pathological process of AD. This model outperformed existing models and demonstrated outstanding diagnostic capabilities. The risk score constructed based on the feature proteins in the model can also distinguish between patients with different degrees of symptoms. Furthermore, metabolite analysis of AD brain tissue and serum confirmed the role of amino acid‐ and lipid‐related metabolic pathways in AD progression.

## 2 Materials and Methods

### 2.1 Data Sources

In this study, multiple datasets were used for analysis, including Mount Sinai/JJ Peters VA Medical Center Brain Bank cohort (MSBB) data, Religious Orders Study and Memory and Aging Project (ROSMAP) data, Alzheimer's Disease Neuroimaging Initiative (ADNI), and Gene Expression Omnibus (GEO) data. MSBB is a protein data sample obtained from the parahippocampal gyrus (*N* = 186, AD = 100, Control = 86). ROSMAP protein data from the dorsolateral prefrontal cortex (*N* = 400, AD = 251, Control = 149) were used for protein‐level validation studies. Metabolic analysis was performed using the metabolite data from the brain and serum of the ROSMAP dataset. GEO data and ANDI data (*n* = 292, AD = 229, Control = 63) were used for validation at the transcriptomic level in the brain tissue and serum. The GEO data included GSE5281 (*N* = 161, AD = 87, Control = 74) [15], GSE1297 (*N* = 31, AD = 22, Control = 9) [16], GSE28146 (*N* = 30, AD = 22, Control = 8), GSE29378 (*N* = 62, AD = 30, Control = 32) [17], GSE118553 (*N* = 309, AD = 231, Control = 78) [18], GSE132903 (*N* = 195, AD = 97, Control = 98) [19], GSE84422 (*N* = 102, AD = 74, Control = 28) [20], GSE122063 (*N* = 100, AD = 56, Control = 44) [21], and GSE48350 (*N* = 253, AD = 80, Control = 173) [22]. MSBB and ROSMAP data were downloaded from Synapse [23]. Download ADNI hematological gene expression data from the ADNI website [24]. GEO data were downloaded from the GEO database [25]. The access and analysis time for all data were January 13, 2024. These data were matched with clinical information and samples without corresponding clinical data were excluded. We obtained the expression matrix of the data and matched the clinical information through the sample ID, such as gender, age, disease status, Braak neurofibrillary tangle staging (Braak), clinical dementia rating (CDR), average amyloid plaque levels (PlaqueMean), etc. Samples with incomplete information are removed in the corresponding analysis. For data processing, the scale and log_2_ functions were used to standardize the data.

### 2.2 Collection of RNA Modification Regulators, Cellular Senescence‐Related Molecules, and AD Risk Genes

RNA modification regulators were collected from published literature [26, 27, 28, 29, 30, 31, 32, 33] and the RM2Target database [34]. The keywords searched included m6A, m1A, m7G, m5C, m5U, m6Am, including 95 RNA modification regulators. The key words “cellular senescence” were searched in Kyoto encyclopedia of genes and genomes (KEGG) [35] and CellAge databases [36] to collect cellular senescence‐related molecules. After removing duplicates, 950 cellular senescence‐related molecules were obtained. The key words “Alzheimer's disease risk genes” were searched through PubMed to obtain AD‐related risk genes from the literature [37, 38, 39] (Table S1).

### 2.3 Identification of Cellular Senescence‐Related Proteins, RNA Modification Regulatory Proteins, and Patient Subtypes Involved in AD Progression

The *K*‐means algorithm in the “cluster” R package identifies patient subtypes. The Elbow method determined the optimal number of clusters to be 2. The “ggplot2” R package was used to visualize the clustering results. The “cor” function in the “Statistics” R package was used to perform Pearson's correlation analysis to assess the relationship between cellular senescence‐related and RNA modification regulatory proteins. Using the Spearman algorithm of the “cor” function, we studied the association between proteins and clinical phenotypes.

### 2.4 Identification of Proteins Involved in AD Progression That Regulate Cellular Senescence‐Related and RNA Modification Regulatory Proteins

Differential expression analysis was performed using the limma package. Benjamini–Hochberg FDR correction was applied to adjust raw *p* values. Proteins with adjusted *p* value (adj.*p*.val) < 0.05 were considered significantly differentially expressed. In order to ensure the scientificalness and robustness of the analysis, this study adopts a differentiated screening strategy. The proteins used for molecular typing, clustering analysis, and screening of differential metabolites were screened by |logFC | > 0, in order to retain the expression change information as complete as possible and avoid the loss of weak but pattern meaningful signals due to strict threshold, so as to ensure the accuracy and stability of clustering results [40, 41, 42]. The characteristic proteins used for machine learning modeling were screened by |logFC | > 0.5 to retain key genes with significant expression differences, clear biological significance and stable signal, reduce noise interference, and improve the generalization ability and prediction performance of the model [43, 44]. Due to the difference analysis based on large‐scale population metabolic samples in this study, there is strong individual heterogeneity in human metabolic profiles. Most functional metabolic differences show weak expression fluctuations, and strict thresholds are easy to filter out key metabolic molecules with potential biological significance. Therefore, this study uses loose screening criteria to initially screen differential metabolites with |logFC| > 0, *p* > 0.05, so as to retain metabolic molecules with differential expression between groups to the greatest extent, avoid missing potentially important metabolic markers related to disease or phenotype, and further screen core effective differential metabolites with enrichment analysis and model screening [45]. A weighted correlation network was constructed using the “weighted gene co‐expression network (WGCNA)” R package [46] and disease‐related modules within the network were assessed. Set the soft threshold to 2 based on the scale‐free topology tit index. Hierarchical clustering and tree analysis were performed using parameters such as mergeCutHeight = 0.25 and minModuleSize = 50 to identify modules containing more than 50 proteins.

### 2.5 Development and Validation of Diagnostic Models

To further identify and simplify AD‐related hub proteins, we used 100 algorithms across 13 machine learning techniques for further screening. The R packages “randomForest,” “xgboost,” “gbm,” “glmnet,” “e1071,” “class,” “tree,” and “MASS” were used to create 13 ML techniques. The “caret” R package was used for cross‐validation and parameter tuning in ML. The dataset was randomly divided into a training set (70%, *n* = 131) and a test set (30%, *n* = 55). An AD diagnostic decision curve was constructed using the “rms” R package by fitting the hub proteins into a binary logistic regression model with a training set.

### 2.6 Interpretation of Model Results by SHapley Additive exPlanations (SHAP) Algorithm

The SHAP algorithm was used to assess the interpretability of the model output [47]. The algorithm provides a measure of feature importance across the entire model and insight into the role of each feature in a specific diagnosis.

### 2.7 Risk Score Construction

To further assess the effect of hub proteins on AD, we constructed a risk score based on 20 hub proteins. Lasso regression was performed with 5‐repeated 10‐fold cross‐validation to determine the optimal regularization parameter (lambda) in a robust manner. The final lambda value was calculated as the mean of lambda.min across five repetitions to ensure stability and reproducibility of feature selection. The optimal *λ* was determined by minimizing binomial bias and the risk score for each patient was calculated by weighing the regression coefficients, as described in the following formula:

$$\text{Risk score}=\sum_{i=1}^{m}x_{i}\cdot \beta_{i}$$

where *X* represents the expression level of the feature protein identified by SHAP and *β* represents the resolution vector of the regression parameter. Methods for constructing risk scores have been applied to patient stratification in conditions such as hepatocellular carcinoma and colorectal cancer [48, 49, 50].

### 2.8 Statistical Analysis

All data processing, statistical analysis, and graphing were performed using R software (v4.4.1/4.5.0). Pearson correlation coefficient was used to assess correlations between continuous variables, while Kruskal–Wallis tests were applied for multiple comparisons. Continuous variables were compared via Wilcoxon rank‐sum test or *t*‐test. For differential proteins expression analysis, the Benjamini–Hochberg (BH) method was used to adjust *p* values for multiple testing, and adjusted *p* value (adj.*p*.val) < 0.05 was considered statistically significant. For correlation analysis and comparisons of gene expression between clusters, thresholds were defined as: *p < 0.05; **p < 0.01; ***p < 0.001; ****p < 0.0001.

## 3 Results

### 3.1 Correlation Analysis of RNA‐Modified Regulators and Cellular Senescence‐Related Proteins With Clinical Features of AD

**FIGURE 1:**
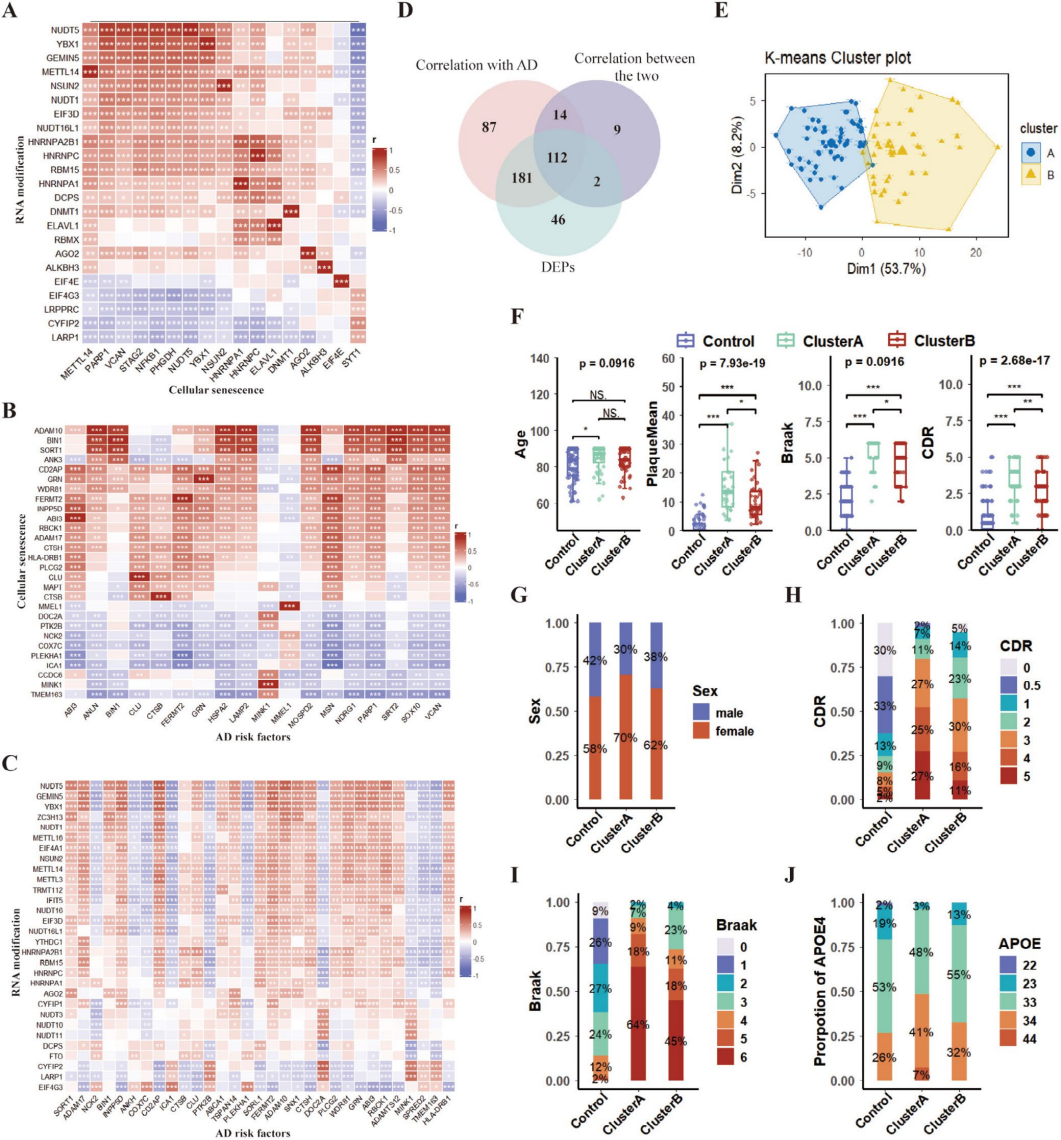
Clustering identifies subtypes of AD patients based on cell senescence‐related proteins and RNA modification regulators. (A) Pearson correlation Heatmap of the RNA modification regulatory proteins and cellular senescence‐related proteins, with a screening threshold of |*r*| > 0.4 and *p* < 0.05. (B, C) Heatmap of Pearson correlations between the AD risk molecules and RNA modification regulatory proteins and cellular senescence‐related proteins, with a correlation threshold set at |*r*| > 0.4 and *p* < 0.05. (D) The Venn diagram shows the intersection of three screening methods for RNA modification regulation and proteins associated with aging: (1) proteins that are differentially expressed in the AD group and control group with |logFC| > 0.5; (2) proteins with a correlation coefficient |*r*| > 0.4 between the two groups; (3) proteins with a correlation coefficient |*r*| > 0.4 with AD risk proteins. A total of 112 common proteins were identified. (E) Based on *K*‐means clustering of 112 shared proteins, AD samples in the MSBB dataset were divided into two subtypes when *K* = 2. (F) Comparison of relevant clinical information (Age, CDR, Braak, and PlaqueMean) between Control, Cluster A, and Cluster B. A *t*‐test was used to detect changes in clinical information between the two groups and a Kruskal–Wallis test was used to detect changes in clinical information among the three groups. *p < 0.05; **p < 0.01; ***p < 0.001; ****p < 0.0001. (G–J) Population distribution ratios between Control, Cluster A, and Cluster B, including gender, Braak, CDR, and APOE genotypes.

To better assess the intrinsic association between cellular senescence and RNA modifications, we performed Pearson's correlation analysis that revealed a strong correlation between RNA modification regulators and cellular senescence‐related proteins (Figure 1A and Table S2). Further evaluation revealed that some of these proteins exhibited significant correlations with AD pathological markers, including Braak staging, CDR scores, and average amyloid plaque levels (Table S3). What's more, they exhibited significant correlations with the expression of AD risk proteins, confirming their close association with AD progression (Figure 1B,C and Table S3).

To assess the specific roles of cellular senescence‐related and RNA modification regulatory proteins at different stages of AD progression, we used clustering algorithms to group patients with AD based on the expression levels of these two types of feature proteins (Figure 1D). In the *K*‐means clustering analysis, the samples exhibited clear distinctions when *K* = 2. We obtained two patient subtypes with different clinical manifestations (Figure 1E). Compared with Cluster B, Cluster A exhibited poorer clinical manifestations regarding Braak, CDR, and PlaqueMean (Figure 1F–J). Based on the above analysis, it is reasonable to speculate that Cluster A may be a subtype that is significantly associated with AD progression.

### 3.2 Exploring Characteristic Molecules That Influenced Disease Progression

**FIGURE 2:**
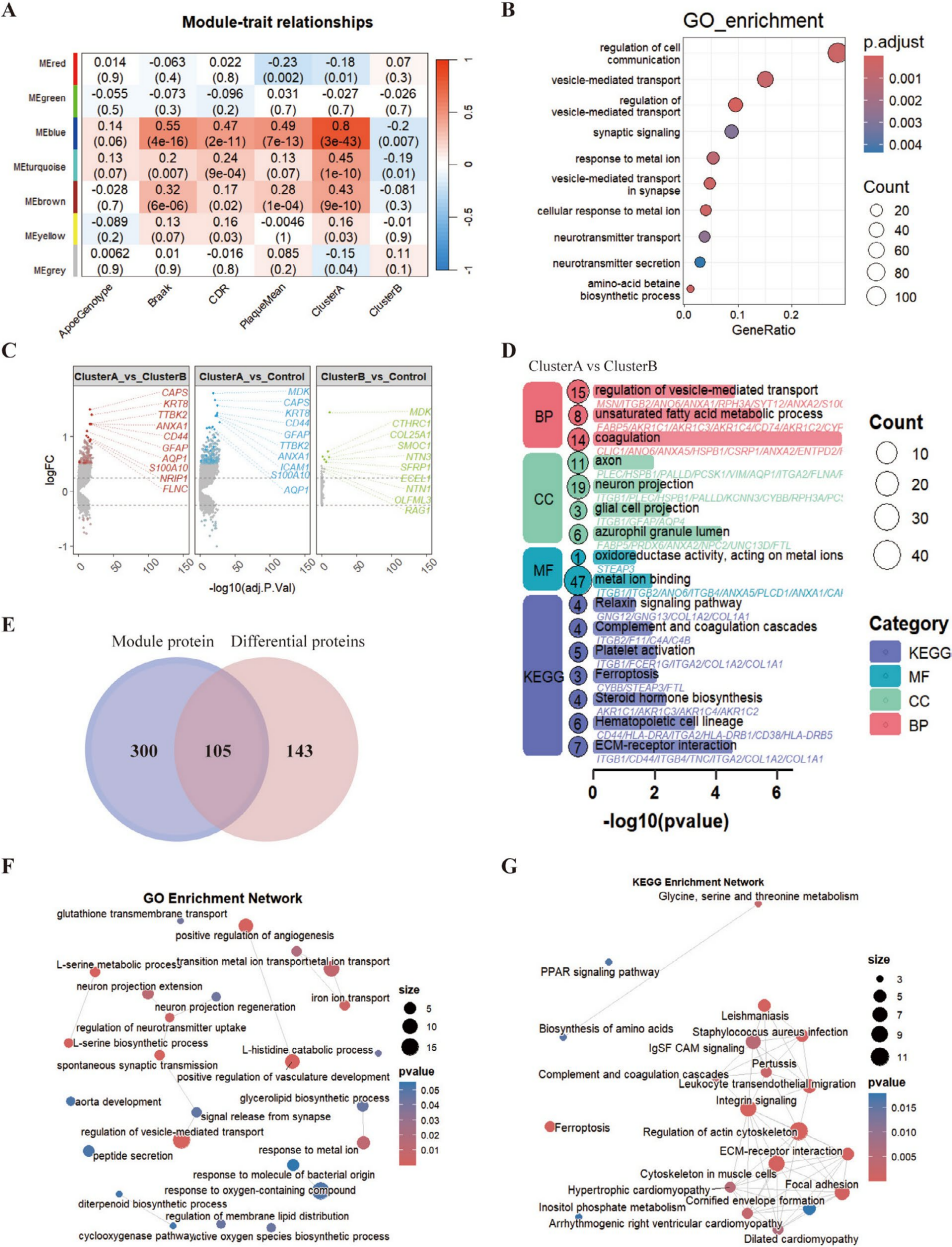
Exploring molecular features related to disease progression. (A) Heatmap showing the correlation between weighted gene co‐expression network modules and Cluster A and Cluster B, along with clinically relevant information. The numbers in parentheses are *p* values, while the numbers outside parentheses indicate the correlation. (B) Dot plot shows the GO enrichment analysis results for the blue‐colored module hub molecules. (C) Differential expression of proteins between Cluster A, Cluster B, and Control was analyzed with the criteria of |logFC| > 0.5 and adj *p* < 0.05. (D) Horizontal bar charts show the GO enrichment pathways and KEGG enrichment pathways of the differential expression analysis of Cluster A and Cluster B. (E) The Venn diagram shows the intersection of WGCNA blue module proteins and differentially expressed proteins, identifying 105 common proteins. (F) The network diagram shows the GO pathway names of the 105 common proteins. (G) The network diagram shows the KEGG pathway names of the 105 common proteins.

According to WGCNA results, we selected a power value of 2 to construct the subsequent analysis module (Figure S1A). We developed a weighted co‐expression network model that divided all proteins into seven modules (Figure S1B). In the module correlation heatmap, the blue module was positively correlated with CDR, Braak, and PlaqueMean (Figure 2A). The module also revealed the highest positive correlation with the cluster A patient subgroup. In combination with the previous analysis results, Cluster A was the subtype most closely associated with AD progression. Therefore, we concluded that the proteins in the blue module were likely to participate in the disease progression of AD and played a certain pathogenic role (Figure S1C). GO enrichment revealed blue module proteins enriched in synaptic vesicle transport, neurotransmitter secretion, and metal ion response. Regulation of cell communication had the highest GeneRatio, gene count, and statistical significance, implying this module modulates AD progression via synaptic function and ion metabolism (Figure 2B). To further study the specific or shared molecules that are vital in various disease processes, we conducted differential analyses (Figure 2C and Figure S1D). In the enrichment analysis of the differentially expressed proteins, we observed changes in metal ion‐related pathways and the ferroptosis pathway. The proteins expression patterns involved in these pathways continued to develop during AD (Figure 2D and Figure S1E,F).

We compared WGCNA analysis results with the differential analysis results of the two subtypes, identifying 105 target proteins (Figure 2E and Table S4). Enrichment analysis of these proteins revealed that they were involved in various amino acid metabolism, lipid metabolism, and metal ion‐related pathways, consistent with the results of our previous study (Figure 2F,G and Table S5).

### 3.3 ML Participated in AD Diagnosis Model Construction

**FIGURE 3:**
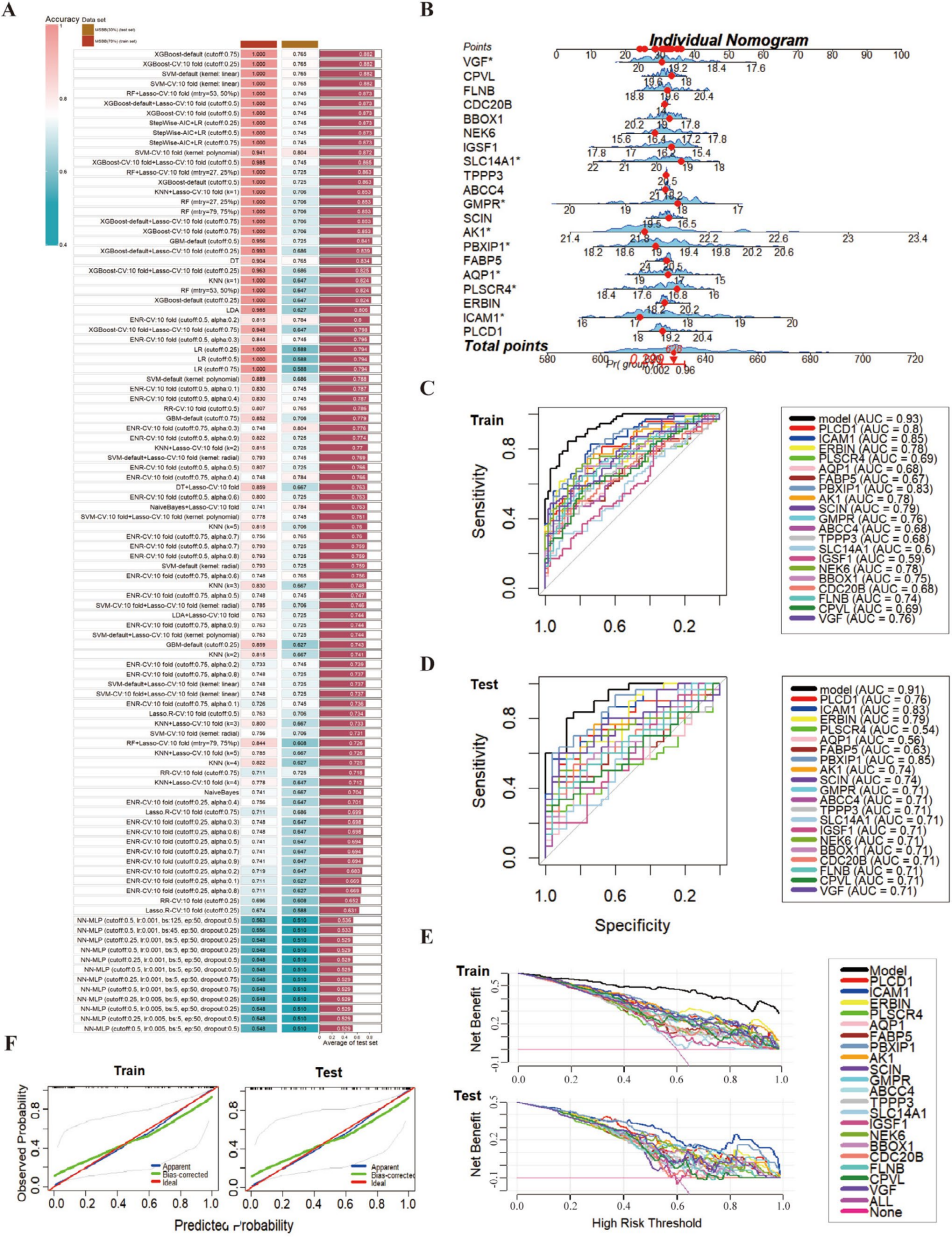
Cross‐machine learning screening of hub proteins. (A) ACC ranking of 100 machine learning algorithm combinations. (B) Diagnostic model constructed using the top 20 hub proteins identified by the XGBoost algorithm, with a dynamic line chart showing the diagnostic status of the 20th sample. (C, D) ROC analysis curves for the 20 hub proteins and the overall model on the internal test set and validation set. (E) Clinical decision curves for the 20 hub proteins and the overall model on the internal test set and validation set. (F) Calibration curves on the internal training set and test set.

To further simplify the set of proteins that may play a pathogenic role in AD, we applied 13 ML algorithms for cross‐validation analysis to screen the 105 proteins obtained from the intersection of the above. XGBoost had an average ACC score of 0.882 in the internal test and validation sets, ranking first with respect to the ACC score (Figure 3A and Figure S2). Next, the top 20 proteins ranked by the Gain index in the XGBoost were extracted, including PLCD1, ICAM1, ERBIN, PLSCR4, AQP1, FABP5, PBXIP1, AK1, SCIN, GMPR, ABCC4, TPPP3, SLC14A1, IGSF1, NEK6, BBOX1, CDC20B, FLNB, CPVL, and VGF (Table S6).

To further assess the diagnostic ability of the 20 proteins for AD, we created a column line graph comprising 20 proteins to assess each sample in detail (Figure 3B). In the training set, the CSRMP model demonstrated high predictive performance, with AUC scores of 0.93 and 0.91 on the internal training set and test set, respectively (Figure 3C,D). The DCA suggested that patients may benefit from the diagnostic model. The clinical benefit of the diagnostic model was higher than the single‐gene curve, indicating that the model can more effectively balance the benefits and treatment risks (Figure 3E). The calibration curve revealed a small error between the diagnostic and actual risks of AD, indicating that the two curves essentially follow the same trajectory, which suggests that the model is highly accurate in predicting AD (Figure 3F).

To compare the diagnostic performance of these features with other labels, we searched the existing literature for 13 sets of diagnostic features for AD [51, 52, 53, 54, 55, 56, 57] and input these features into a diagnostic model to compare the AUC scores of these 13 sets of features in GEO, ROSMAP, and ADNI datasets. In summary, the features selected based on RNA modifications and cellular senescence can be validated at the protein and transcriptome levels, signifying a diagnostic model and feature set stability (Figure S3 and Table S6).

### 3.4 Constructing and Evaluating Patient Risk Scores Based on Hub Proteins

**FIGURE 4:**
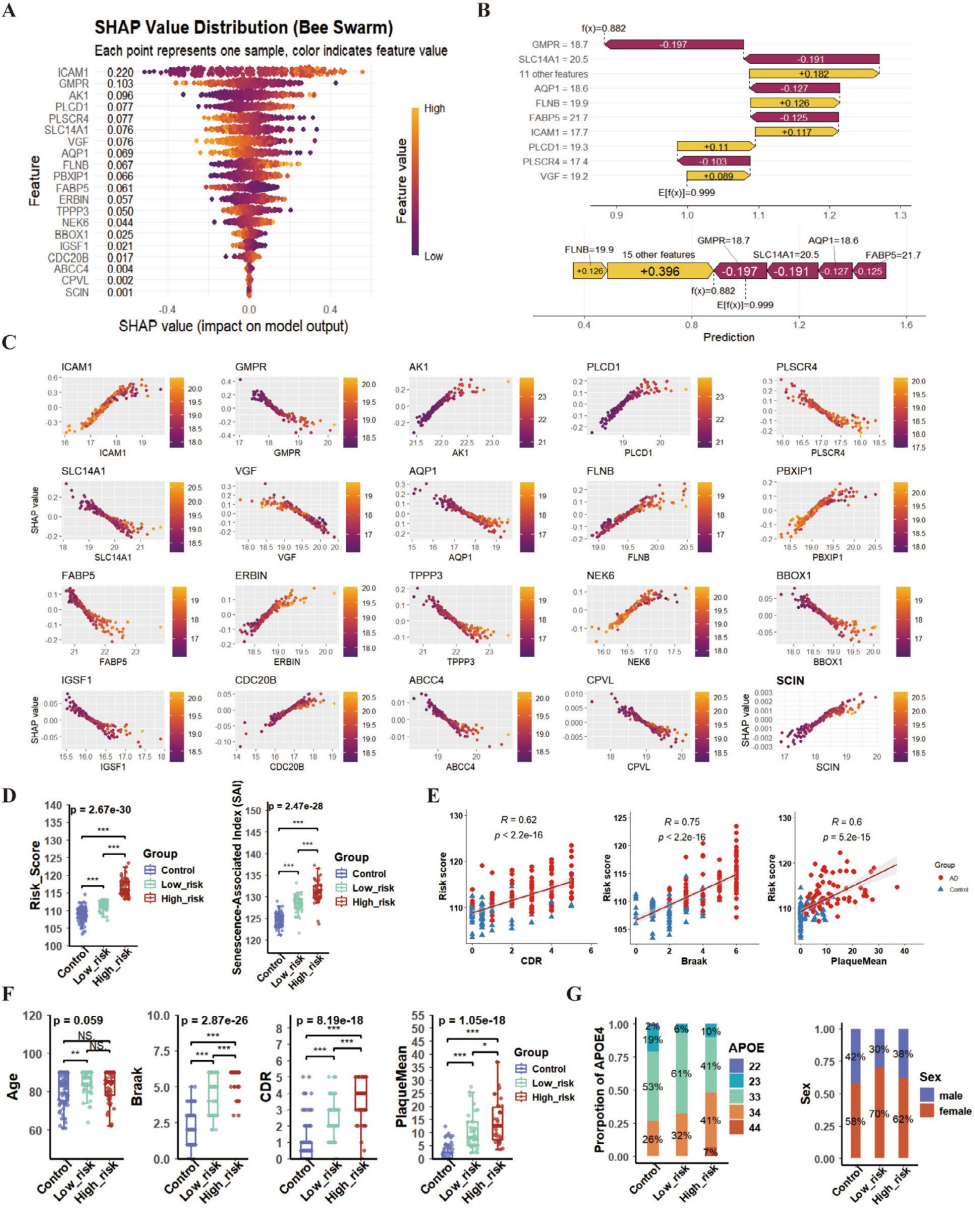
Construction of patient risk scores. (A) The beeswarm plot shows the SHAP averages to evaluate the contribution of features to the model. (B) The force plot and waterfall plot show the role of each feature in the prediction of the 20th sample, helping to understand the model's decision‐making process. (C) Dependency graphs reveal nonlinear relationships between individual features and predictions. (D) Box plots show the difference in risk scores and SAI scores between High_risk, Low_risk, and Control. *T*‐tests were used to compare continuous variables between two independent groups. *p < 0.05; **p < 0.01; ***p < 0.001; ****p < 0.0001. (E) Linear regression analysis of the correlation between clinical manifestations of the disease (CDR, Braak, and PlaqueMean) and risk scores. (F) Comparison of relevant clinical information (Age, CDR, Braak, and PlaqueMean) between Control, high‐risk patients, and low‐risk patients. *T*‐tests were used to detect changes in clinical information between two groups and Kruskal–Wallis tests were used to detect changes in clinical information among three groups. *p < 0.05; **p < 0.01; ***p < 0.001; ****p < 0.0001. (G) Population distribution ratios between high‐risk and low‐risk patients in the control group, including gender, Braak stage, CDR, and APOE genotype.

The SHAP algorithm was used to provide global and local explanations of the model. The top 10 proteins with the greatest contribution to the model were ICAM1, GMPR, AK1, PLCD1, PLSCR4, SLC14A1, VGF, AQP1, FLNB, and PBXIP1 (Figure 4A). Force and waterfall plots exhibit the SHAP values of the 20th sample and their impact on the model diagnosis results (Figure 4B). Among the 20 proteins, ICAM1, AK1, PLCD1, FLNB, ERBIN, NEK6, CDC20B, PBXIP1, and SCIN were positively correlated with the SHAP values. GMPR, SLC14A1, VGF, AQP1, FABP5, TPPP3, IGSF1, ABCC4, PLSCR4, BBOX1, and CPVL were negatively correlated with SHAP values (Figure 4C). Meanwhile, 20 proteins also showed significant correlations with cellular senescence and RNA modifications (Figure S4A,B).

To further evaluate the overall role of these 20 proteins in AD pathology and elucidate the underlying molecular mechanisms, we calculated the risk score for each patient based on the expression values of the 20 proteins weighted by their regression coefficients (Table S7). We observed significant differences in risk scores, senescence‐associated index, and expression of key proteins between control and AD patients (Figure 4D and Figure S4C). In addition, the high‐risk group had the highest senescence‐associated index (SAI) scores, suggesting that senescence‐associated molecules may be vital for the disease process but may not necessarily be reflected in actual age differences (Figure 4D). The median risk score was used as the optimal cutoff value to divide all patients into high‐ and low‐risk groups. Correlation analysis revealed that the risk score was highly positively correlated with CDR, Braak, and PlaqueMean (Figure 4E and Figure S5). Twenty hub proteins exhibited significant differences among the three groups in the assessment of CDR, Braak, and PlaqueMean (Figures S6–S8). The high‐risk group had the highest CDR, Braak, and PlaqueMean, representing more obvious disease characteristics (Figure 4F,G). In the application of the risk score in external independent datasets, we observed a trend that individuals in the high‐risk group had higher pathological scores and lower cognitive scores, along with a greater probability of APOE4 carriage and a higher proportion of female subjects (Figure S9A,B).

### 3.5 Metabolic Protein Level Analysis Based on High‐ and Low‐Risk Groups

**FIGURE 5:**
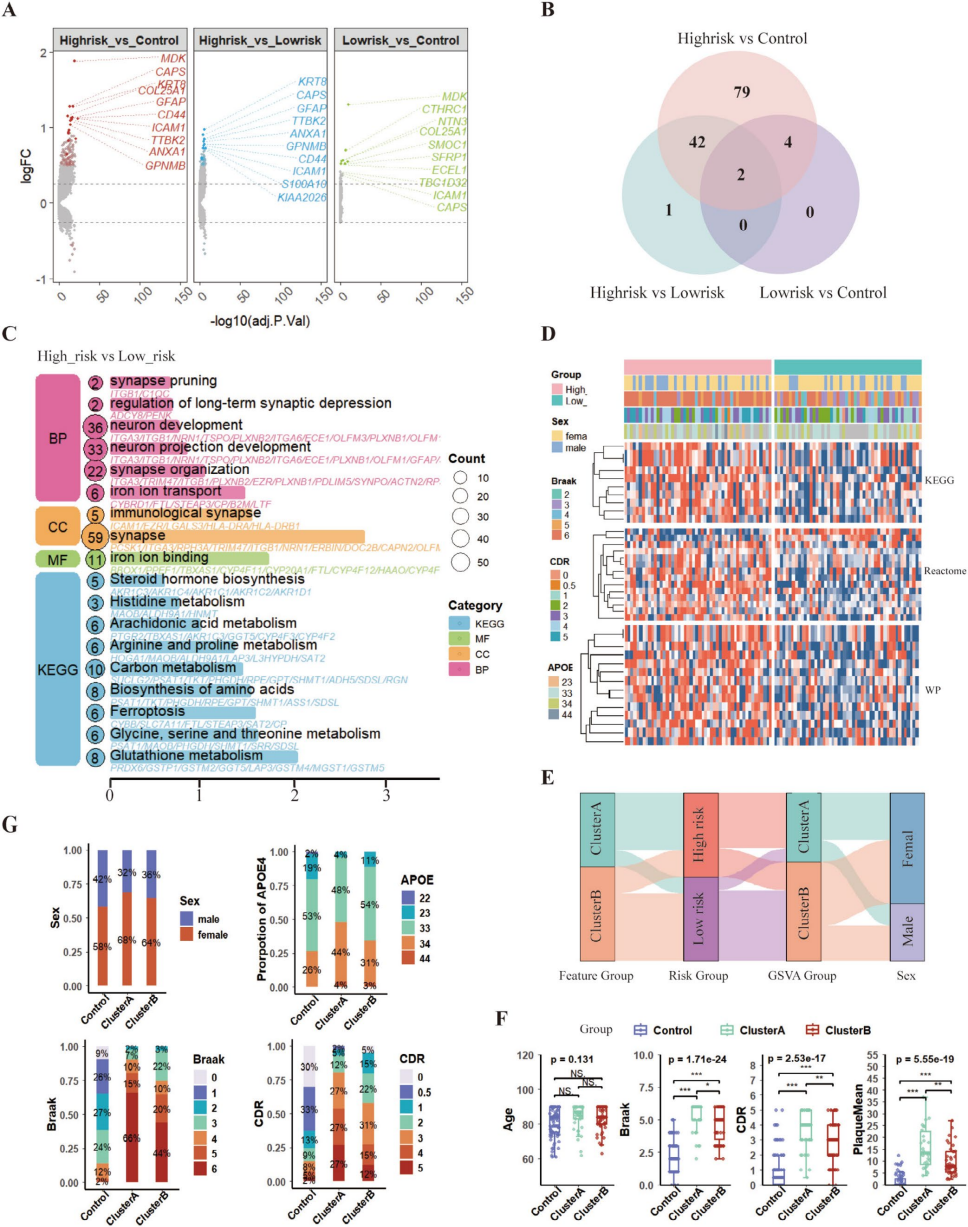
Metabolic analysis of protein levels based on high‐risk and low‐risk grouping. (A) Protein differential expression analysis between control, high‐risk, and low‐risk patients, |logFC| > 0.5, adj *p* < 0.05. (B) Venn diagram showing the intersection of protein differential analysis results. (C) Horizontal bar charts showing GO pathways and KEGG pathways enriched with proteins that differ between high‐risk and low‐risk groups. (D) Heatmap shows the differences in KEGG, REACTOME, and WiPi metabolic pathways based on GSVA scores for sample patients. (E) Sankey Diagram shows the population ratio of high‐risk and low‐risk patients according to GSVA scores. (F) Comparison of clinical information related to Control and GSVA subtypes. *T*‐tests were used to detect changes in clinical information between the two groups and Kruskal–Wallis tests were used to detect changes in clinical information among the three groups. *p < 0.05; **p < 0.01; ***p < 0.001; ****p < 0.0001. (G) Population distribution proportions of control and metabolic subtype groups, including gender, Braak stage, CDR, and APOE genotype.

In this study, we identified many metabolism‐related pathways from the enrichment analysis of 105 proteins. Consistent with this study, abnormalities in certain metabolic pathways have indeed been observed in neurodegenerative diseases [14, 58, 59]. To further identify the potential molecular mechanisms of the group primarily regulated by hub proteins, we performed differential analysis of high‐risk and low‐risk groups (Figure 5A,B). GO and KEGG analysis revealed enrichment in numerous neuro‐related pathways (Figure 5C). The neuron projection development pathway was also considered a pathological pathway specific to female patients with AD [60]. Metabolism‐related pathways and metal ion‐related pathways were also enriched between high‐ and low‐risk groups (Table S8). These results demonstrate the key role of metal ion transport and metabolic dysfunction in AD progression.

To further understand the dynamic changes in metabolic pathways during AD progression, we performed GSVA metabolism‐related analysis. Differential analysis of GSVA scores of metabolic pathways between high‐ and low‐risk groups revealed that numerous lipid and amino acid metabolism pathways were activated in the high‐risk group (Figure 5D and Table S9). We applied cluster analysis to group patient samples following their GSVA scores, obtaining two metabolism‐related subtypes: GSVA_Cluster A and GSVA_Cluster B (Figure 5E). Regarding clinical manifestations, GSVA_Cluster B was closer to the low‐risk group, whereas GSVA_Cluster A was closer to the high‐risk group (Figure 5F,G and Table S10).

### 3.6 Metabolite Analysis of the Brain and Serum of Patients With AD

**FIGURE 6:**
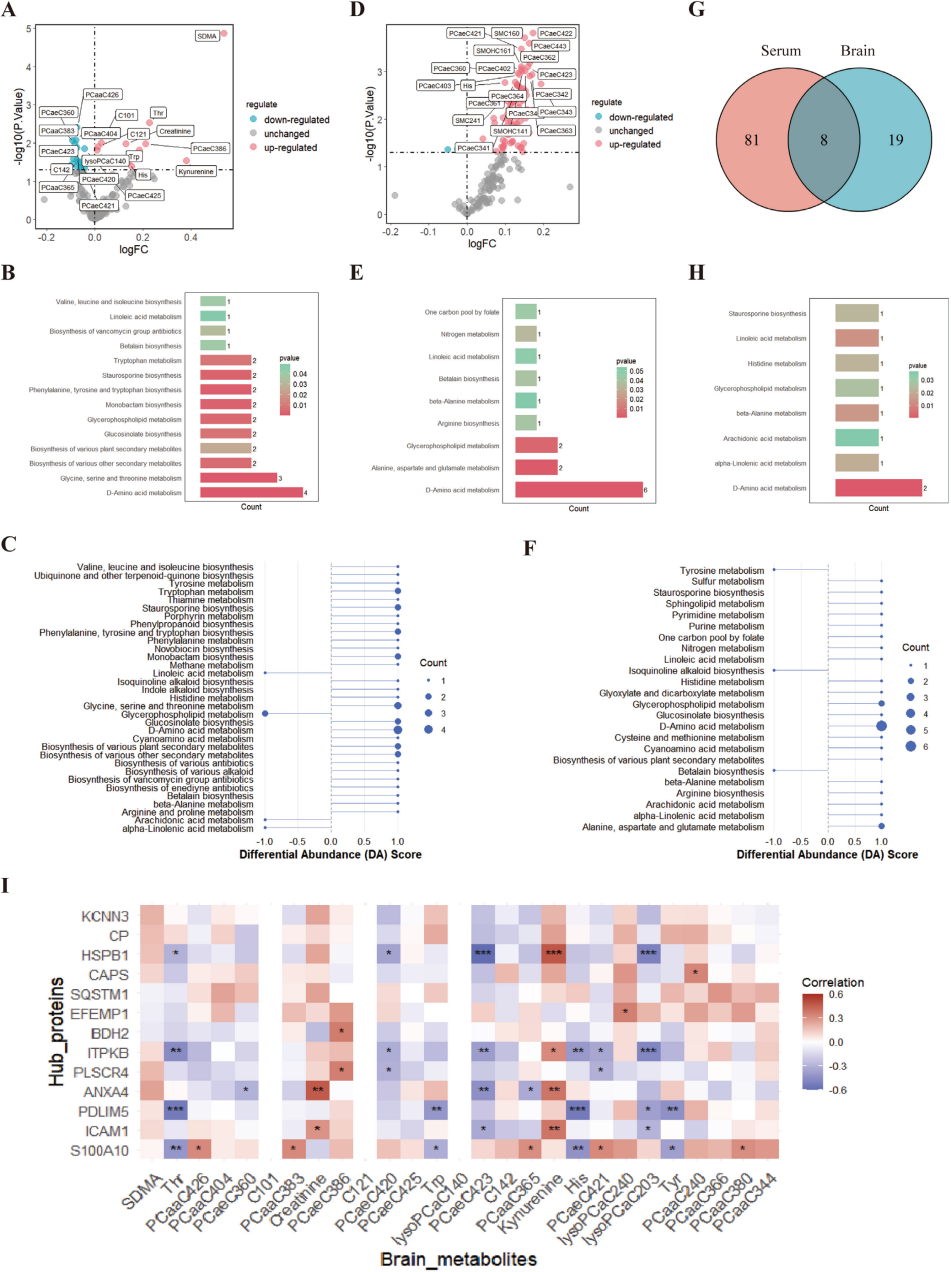
Analysis of metabolites in the brain and serum. (A) Differential analysis of metabolites in the brain, with selection criteria of |logFC| > 0 and *p* < 0.05. (B) KEGG enrichment analysis of differentially expressed metabolites in the brain. (C) DA abundance analysis of differentially expressed metabolites in the brain. (D) Differential analysis of metabolites in serum, with selection criteria of |logFC| > 0 and *p* < 0.05. (E) KEGG enrichment analysis of differentially expressed metabolites in serum. (F) DA abundance analysis of differentially expressed metabolites in serum. (G) Differential metabolites common to both brain and serum. (H) KEGG metabolic pathways enriched in differentially expressed metabolites common to both brain and serum. (I) Correlation heatmap showing the correlation analysis between differentially expressed metabolites in the brain and hub proteins, *p < 0.05; **p < 0.01; ***p < 0.001; ****p < 0.0001.

Previous studies have reported abnormal changes of serum metabolites in the course of AD disease [61, 62]. To further investigate the patterns of brain and serum metabolite changes in AD, we performed differential metabolite analysis on brain tissue and serum samples to uncover cross‐tissue metabolic differences. Through differential analysis of metabolites, we identified 27 differentially expressed metabolites, of which 11 were upregulated and 16 were downregulated in the brain tissue (Figure 6A). KEGG enrichment analysis revealed abnormal activity in amino acid and phospholipid‐related metabolism in brain tissue (Figure 6B). Differential abundance (DA) analysis was employed to evaluate the average values and overall changes of metabolites involved in corresponding metabolic pathways, which depicted that metabolic pathways, including D‐amino acid metabolism, exhibited an overall trend of upregulation (Figure 6C). This was similar to our previous metabolic research results based on protein levels. Metabolite difference analysis in the serum included 86 upregulated and 2 downregulated metabolites (Figure 6D). Similar to brain metabolites, the metabolic analysis of peripheral blood also revealed changes in Betalain biosynthesis, D‐amino acid metabolism, and glycerophospholipid metabolism (Figure 6E,F). Comparing the results of the brain tissue and serum differential analysis, there were eight common differential metabolites, mainly histidine and phosphatidylcholine metabolites (Figure 6G,H and Table S11).

There was a certain relationship between brain hub proteins and metabolites (Figure 6I). Metabolites, including threonine, PCaaC423, kynurenine, and lysoPCaC203, were correlated with more than four hub proteins, suggesting that these metabolites may be vital for the metabolic regulatory network in the brain through interactions with key proteins. Conversely, the correlation between the metabolites and serum hub proteins was weaker (Figure S10A). Further analysis of the correlation between the clinical manifestations and metabolites revealed that the mini‐mental state examination (MMSE) score was significantly associated with certain differential metabolites (Figure S10B,C), suggesting that abnormal changes in these metabolites may be closely associated with cognitive impairment in AD.

## 4 Discussion

Current findings suggested that RNA modification and cellular senescence play a role in the progression of AD [63, 64]. We compiled common RNA modification regulatory proteins from previous studies, including m6A, m1A, m5C, m5U, m6Am, and m7G. To further study the combined effects of these six RNA modifications and cellular senescence in AD, we first screened characteristic proteins with strong correlations for subsequent analysis. According to the expression levels of the proteins, patients were divided into two subtypes. The correlation analysis between subtypes and clinical manifestations of AD can help us screen for patient subtypes that are closely associated with AD progression. It was observed that Cluster A subtypes exhibited high values in pathological indicators, as reflected in Braak staging closer to the terminal stage of neurodegeneration, CDR scores corresponding to more severe cognitive decline and PlaqueMean reflecting a significantly higher amyloid plaque burden in the brain. This indicates that RNA modification and cellular senescence are key drivers of AD progression [65, 66, 67].

Next, we used 13 types of machine learning cross‐calculations to simplify the key proteins, identify 20 hub proteins and use them to construct a diagnostic model. The risk score calculated using the regression coefficient‐weighted expression of 20 proteins can effectively assess the severity of conditions of patients with AD and the proportion of high‐risk individuals overlapped significantly with ClusterA, highlighting the potential key role of hub proteins. Finally, we applied GSVA analysis of proteins, differential metabolite enrichment analysis and DA analysis of metabolites to investigate the metabolic subtypes and metabolic pathway characteristics during AD progression. The results revealed that GSVA_ClusterA was more similar to the distribution of high‐risk populations corresponding to changes in metabolic characteristics related to amino acids and lipids. Our study is the first to integrate 6 RNA modifications regulators and cellular senescence‐related proteins to investigate their combined impact on AD progression, providing a new perspective for understanding the mechanisms of AD.

When performing enrichment analysis on the results of the three‐group difference analysis for Control and Clusters A and B, we identified pathways associated with iron metal ions, specifically including “response to metal ion” and “iron ion transport” pathways. The pathways related to ferroptosis have also been identified. Based on a previous study, senescent cells can accumulate numerous intracellular iron, which is attributed to impaired ferritin phagocytosis and ferroptosis suppression [68]. While our feature set maintains dominance in GEO datasets, the ferroptosis feature set occasionally achieves superior performance when comparing AUC scores across gene sets. Transcriptomes reflect gene transcriptional activity, whereas proteins are direct carriers of functional performance. The expression patterns of the two are not entirely consistent and this dimensional mismatch may weaken the diagnostic efficacy of our feature set, making the ferroptosis feature set more suited to diagnostic needs in specific data environments.

The results indicating that our research findings have a certain application potential in clinical practice and may provide a reference for disease diagnosis or condition assessment. Apart from VGF, 19 proteins were upregulated in patients with high‐risk scores, reflecting the overall contribution of these proteins to the risk score. Beckmann et al. [69] have confirmed VGF as a key regulator in the progression of AD through multiscale coronary networks. Studies found that VGF can reduce microglial inflammation, repair synaptic plasticity, and alleviate pathological cognition in mice [70, 71]. NEK6, CDC20B, PBXIP1, and SLC14A1 have been identified as diagnostic biomarkers of AD in cerebrospinal fluid or brain tissue [72, 73, 74, 75]. Pathologically upregulated FABP5 serves as a vital marker and regulator of ferroptosis [76]. This trend aligns with our subsequent ferroptosis‐related findings. Accordingly, aberrant RNA modification and cellular senescence may drive AD progression by triggering ferroptosis. In addition, SQSTM1 is an autophagy‐related protein, β‐amyloid and tau protein accumulation impair autophagy, which further promotes the accumulation of toxic substances and cellular senescence, forming a vicious cycle [77]. Therefore, targeting autophagy and stabilizing autophagic function may be potential strategies for simultaneously intervening in cellular senescence and AD progression. These molecules in glial cells are vital for maintaining glial cell function in the brains of patients with AD, providing a theoretical basis for understanding the pathological network and therapeutic targets of AD.

In the metabolite analysis results, we observed that histidine level was upregulated in the brains and serum of AD patients. In a Mendelian randomization study, histidine had a causal effect on the risk of AD [61, 78]. The combined analysis of protein metabolism and metabolites can explicate the functional state of biological systems from the dual dimensions of “metabolic processes” and “metabolic end products.” Based on the protein metabolic analysis, our attention focused on some pathways. “Positive regulation of macromolecule metabolic process” pathway includes five proteins, YBX1, HNRNPC, METTL14, HNRNPA1, and DNMT1, which are upregulated in the AD state. HNRNPC and HNRNPA1 participate in the splicing process by recognizing m6A, thereby affecting lipid metabolism during aging or disease states [79, 80]. METTL14, a writer component of m6A modification, forms a functional complex with METTL3 to catalyze m6A generation. Zhang et al. [81] indicate that m6A RNA modification is involved in various metabolic pathways and the development of common metabolic diseases, making it a potential disease‐specific therapeutic target.

We observed that the phosphatidylcholine metabolite level in the brain and serum of patients is inconsistent. Among the 20 key proteins, ICAM‐1 upregulation showed increased blood–brain barrier permeability [82]. Additionally, the expression of astrocyte activation markers GFAP (UniProt P14136) and S100B (UniProt P04271) was upregulated in high‐ and low‐risk groups. The abnormal expression of these BBB‐specific proteins and differential expression of metabolites may suggest that the structure of the BBB may change during AD progression [83, 84]. AQP1 is a selective transmembrane aquaporin. BBB breakdown elevates AQP4 expression. Persistent AQP4 overexpression leads to intracellular water overload, cytotoxic edema, and aggravated brain injury [85].

The core findings of this study are primarily based on bioinformatics analysis or data derived from model organisms. The specific biological functions of the target proteins have not yet been confirmed through functional validation methods such as in vitro cell experiments or in vivo animal models. Furthermore, the study lacks support from large‐scale, multicenter clinical cohort data, and the prevalence and specificity of these findings in AD patients require further validation. AD is a highly heterogeneous disease, exhibiting significant individual variation in age of onset, clinical phenotypes, and pathological subtypes. This study has not sufficiently incorporated stratified analysis of these heterogeneity factors in data analysis or mechanism exploration; further investigation is needed into the differences in molecular regulatory networks underlying individual variations.

## 5 Conclusions

We integrated RNA modification regulators and cellular senescence‐related proteins to explore the biological pathways and molecular co‐expression networks affected by pathological processes in different AD subtypes under their coordinated regulation. Based on this, we incorporated transcriptomic data to construct and validate diagnostic models for assessing AD pathological progression, providing potential tools for early disease detection and progression monitoring. Multilevel validation analysis further revealed metabolic disorder in AD patients, offering a novel research perspective and theoretical basis for elucidating the complex molecular pathogenesis of AD and identifying potential therapeutic targets.

## Author Contributions

Conception and design: D.W., Q.X., and F.L. Administrative support: X.C., L.L., X.H., T.Z., and X.G. Collection and assembly of data: M.T. and K.Y. Data analysis and interpretation: M.T. and K.Y. Manuscript writing: M.T. All authors read and approved the final manuscript.

## Funding

The authors have nothing to report.

## Ethics Statement

The samples included in this study were drawn from publicly available datasets. Relevant ethical approval was obtained for each cohort and informed consent was received from all participants prior to participation. Therefore, no additional ethical approval was required for this study.

## Conflicts of Interest

The authors declare no conflicts of interest.

## Supporting information


**Figure S1:** WGCNA and differential analysis identify disease‐related proteins. (A) Soft threshold screening of gene co‐expression networks, with a soft threshold of two selected based on *R* ^2^ > 0.85 and average connectivity. (B) WGCNA clustering dendrogram. (C) Screening of proteins in the blue module based on PS ≥ 0.4 and MM ≥ 0.4. (D) Intersection of differentially expressed proteins between Cluster A, Cluster B, and the control group in two comparisons. (E) GO and KEGG enrichment analysis of differentially expressed proteins between Cluster B and the control group. (F) GO and KEGG enrichment analysis of differentially expressed proteins between Cluster A and the control group.

**Figure S2:** Ranking of *F*‐score for 100 machine learning algorithms.

**Figure S3:** AUC ranking of different feature combinations. AUC scores of SenoRNA_modifiers and 13 published signatures set in MSBB (*n* = 186), ROXMAP (*n* = 400), GSE5281 (*n* = 161), GSE1297 (*n* = 31), GSE28146 (*n* = 30), GSE29378 (*n* = 62), GSE118553 (*n* = 309), GSE132903 (*n* = 195), GSE84422 (*n* = 102), GSE122063 (*n* = 100), GSE48350 (*n* = 253), ADNI (*n* = 292).

**Figure S4:** Correlation of key proteins with cellular senescence and RNA modification. (A) Correlation heatmap showing the correlation between the 20 hub proteins and proteins related to cellular senescence. (B) Correlation heatmap showing the correlation between the 20 hub proteins and RNA modification regulatory proteins. (C) Box plots showing expression changes of 20 hub proteins in the control, high‐risk, and low‐risk groups. *p* value: * < 0.05; ** < 0.01; *** < 0.001; **** < 0.0001.

**Figure S5:** Linear regression analysis of 20 hub proteins and risk scores.

**Figure S6:** The linear regression analysis of 20 hub proteins and Braak scores.

**Figure S7:** The linear regression analysis of 20 hub proteins and CDR scores.

**Figure S8:** The linear regression analysis of 20 hub proteins and PlaqueMean scores.

**Figure S9:** Correlation analysis of clinical indicators in high‐ and low‐risk groups from external datasets. (A) Proportion of population distribution in control, high‐ and low‐risk groups, including age, gender, Braak, and APOE genotypes in GSE48350. (B) Proportion of population distribution in control, high‐ and low‐risk groups, including age, gender, Braak, and APOE genotypes in GSE29378.

**Figure S10:** Correlation analysis of differentially expressed metabolites in brain and serum. (A, B) Correlation heatmap of serum differential metabolites, hub proteins, and clinical indicators, *p* value: * < 0.05; ** < 0.01; *** < 0.001; **** < 0.0001. (C) Correlation heatmap showing the correlation analysis between differentially expressed metabolites in the brain and clinical manifestations of the disease (sex, APOE genotype, MMSE, Braak). *p* value: * < 0.05; ** < 0.01; *** < 0.001; **** < 0.0001.

**Table S1:** Molecules list of cellular senescence and RNA modification regulatory molecules and AD risk factors.

**Table S2:** List of molecules strongly associated with AD in cellular senescence and RNA modification.

**Table S3:** Correlation analysis of protein on AD pathology.

**Table S4:** Intersection proteins of WGCNA and differential expression analysis.

**Table S5:** Enrichment analysis of WGCNA hub proteins and intersection proteins.

**Table S6:** AUC scores for the top 20 protein features and other predicted features.

**Table S7:** Protein coefficient and risk stratification.

**Table S8:** Differential expression and enrichment analysis of high‐risk and low‐risk groups.

**Table S9:** Difference analysis of GSVA score in AD patients.

**Table S10:** Comparison of three types of patient populations in clusters.

**Table S11:** Differential analysis and enrichment analysis of brain and serum metabolites.

## Data Availability

The data that support the findings of this study are available from the corresponding author upon reasonable request.
