# Supplementary figures and images for: Multilayer Proteome and Metabolome‐Based Validation Uncovers Combined Regulatory Roles and Predictive Values of 6 RNA Modifications and Cellular Senescence in Alzheimer's Disease
Source: CNS Neurosci Ther. 2026 Jul 14;32(7):e71021. doi: 10.1002/cns.71021 (PMC13367321; doi:10.1002/cns.71021)

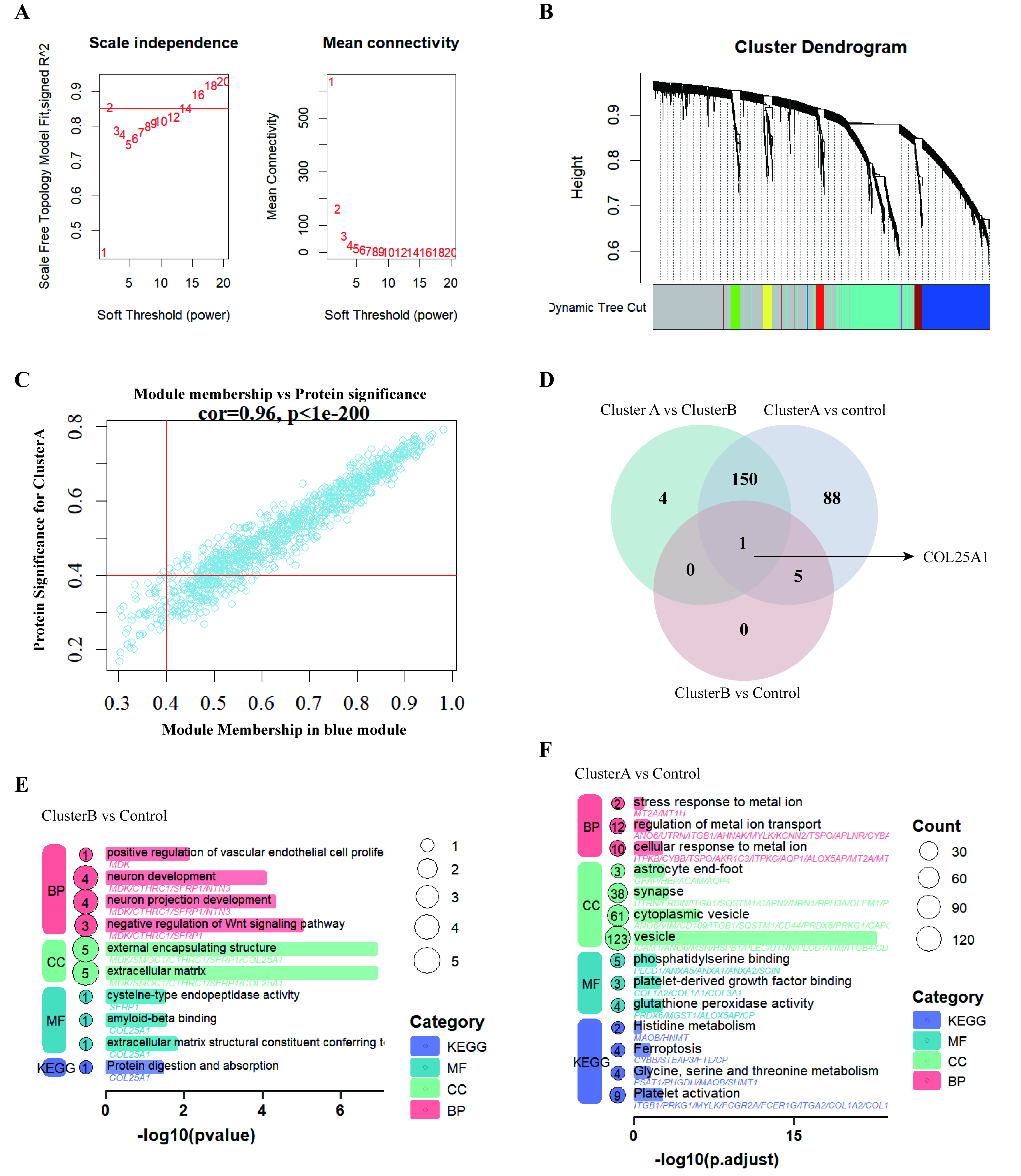

Supplement: Supplementary file 1 — Figure S1: WGCNA and differential analysis identify disease‐related proteins. (A) Soft threshold screening of gene co‐expression networks, with a soft threshold of two selected based on R 2 > 0.85 and average connectivity. (B) WGCNA clustering dendrogram. (C) Screening of proteins in the blue module based on PS ≥ 0.4 and MM ≥ 0.4. (D) Intersection of differentially expressed proteins between Cluster A, Cluster B, and the control group in two comparisons. (E) GO and KEGG enrichment analysis of differentially expressed proteins between Cluster B and the control group. (F) GO and KEGG enrichment analysis of differentially expressed proteins between Cluster A and the control group. [file CNS-32-e71021-s019.tif]

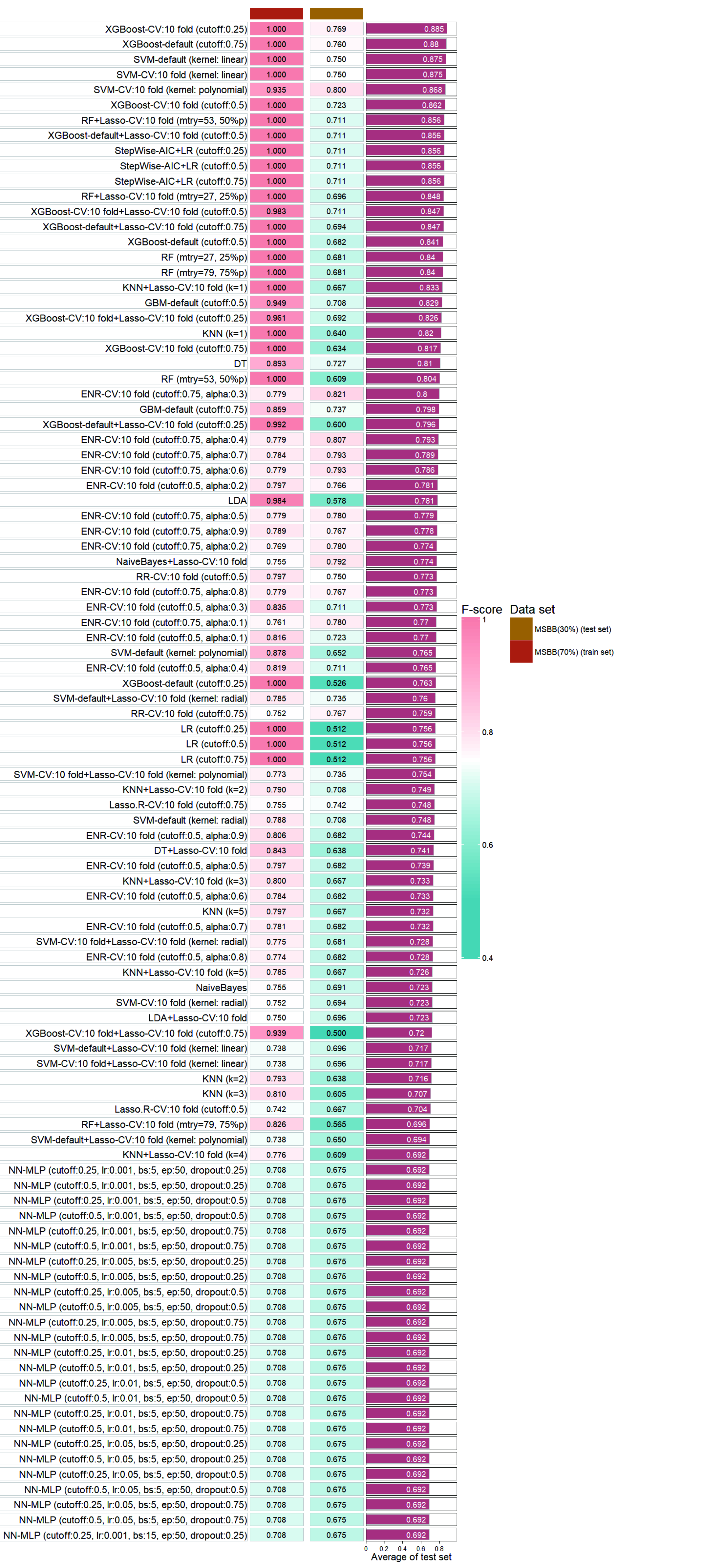

Supplement: Supplementary file 2 — Figure S2: Ranking of F‐score for 100 machine learning algorithms. [file CNS-32-e71021-s017.tif]

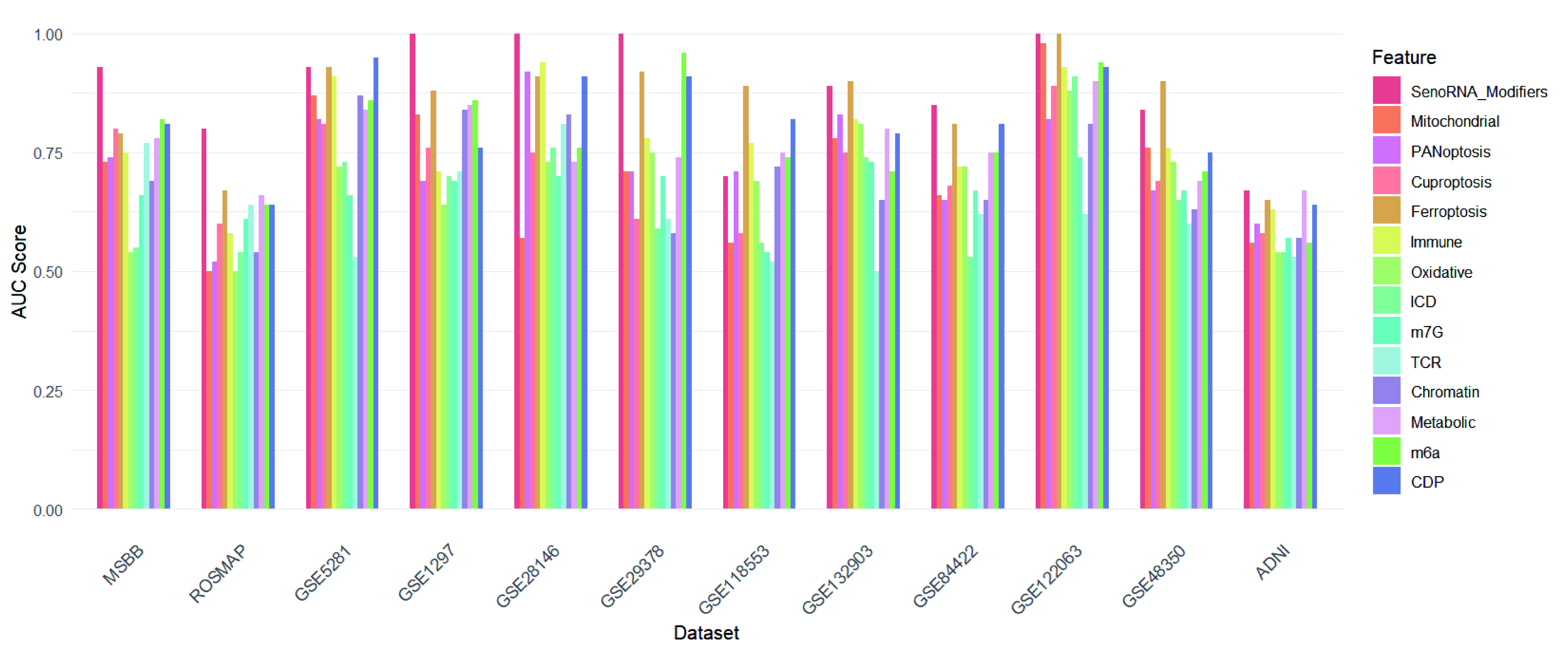

Supplement: Supplementary file 3 — Figure S3: AUC ranking of different feature combinations. AUC scores of SenoRNA_modifiers and 13 published signatures set in MSBB (n = 186), ROXMAP (n = 400), GSE5281 (n = 161), GSE1297 (n = 31), GSE28146 (n = 30), GSE29378 (n = 62), GSE118553 (n = 309), GSE132903 (n = 195), GSE84422 (n = 102), GSE122063 (n = 100), GSE48350 (n = 253), ADNI (n = 292). [file CNS-32-e71021-s004.tif]

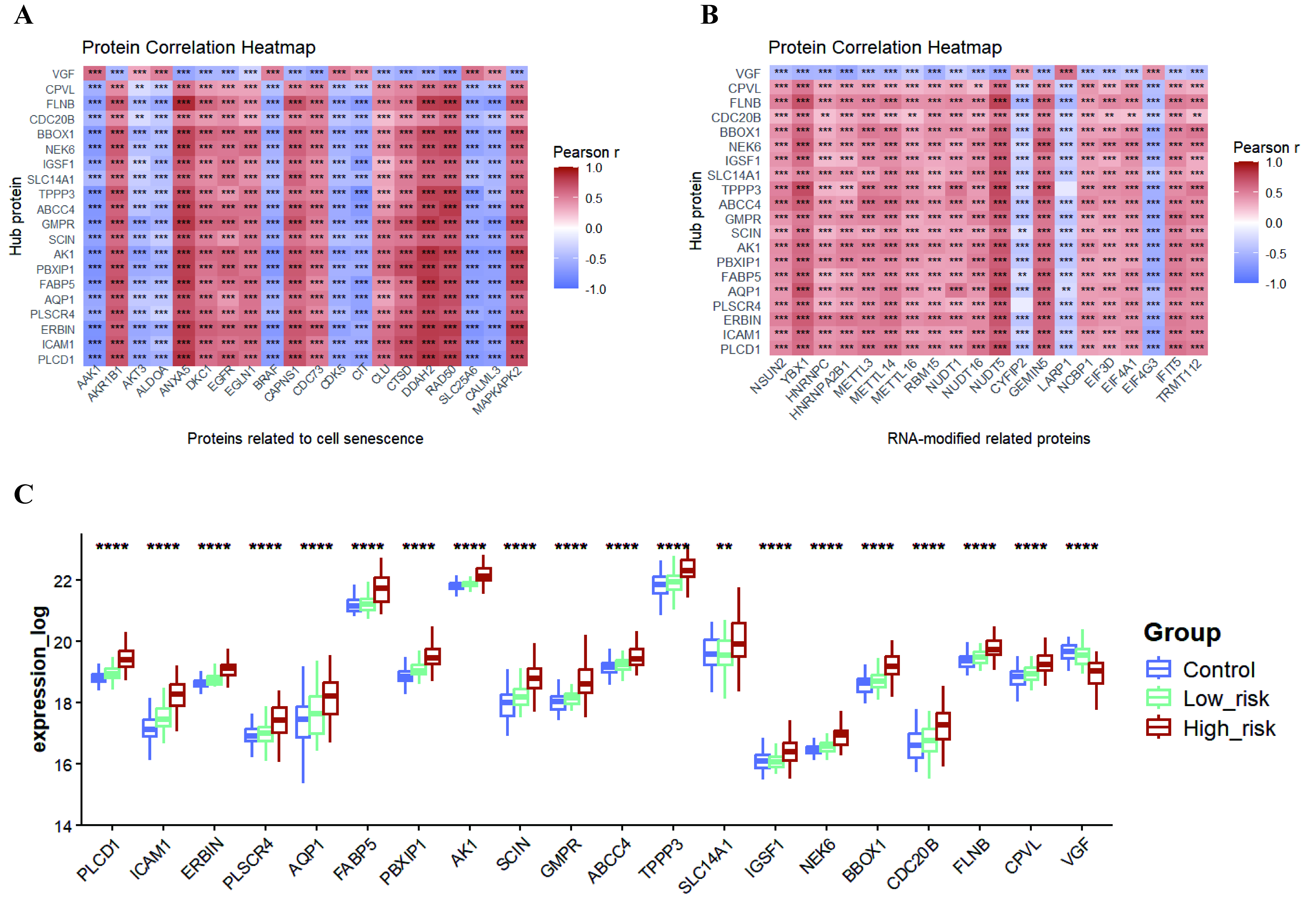

Supplement: Supplementary file 4 — Figure S4: Correlation of key proteins with cellular senescence and RNA modification. (A) Correlation heatmap showing the correlation between the 20 hub proteins and proteins related to cellular senescence. (B) Correlation heatmap showing the correlation between the 20 hub proteins and RNA modification regulatory proteins. (C) Box plots showing expression changes of 20 hub proteins in the control, high‐risk, and low‐risk groups. p value: * < 0.05; ** < 0.01; *** < 0.001; **** < 0.0001. [file CNS-32-e71021-s013.tif]

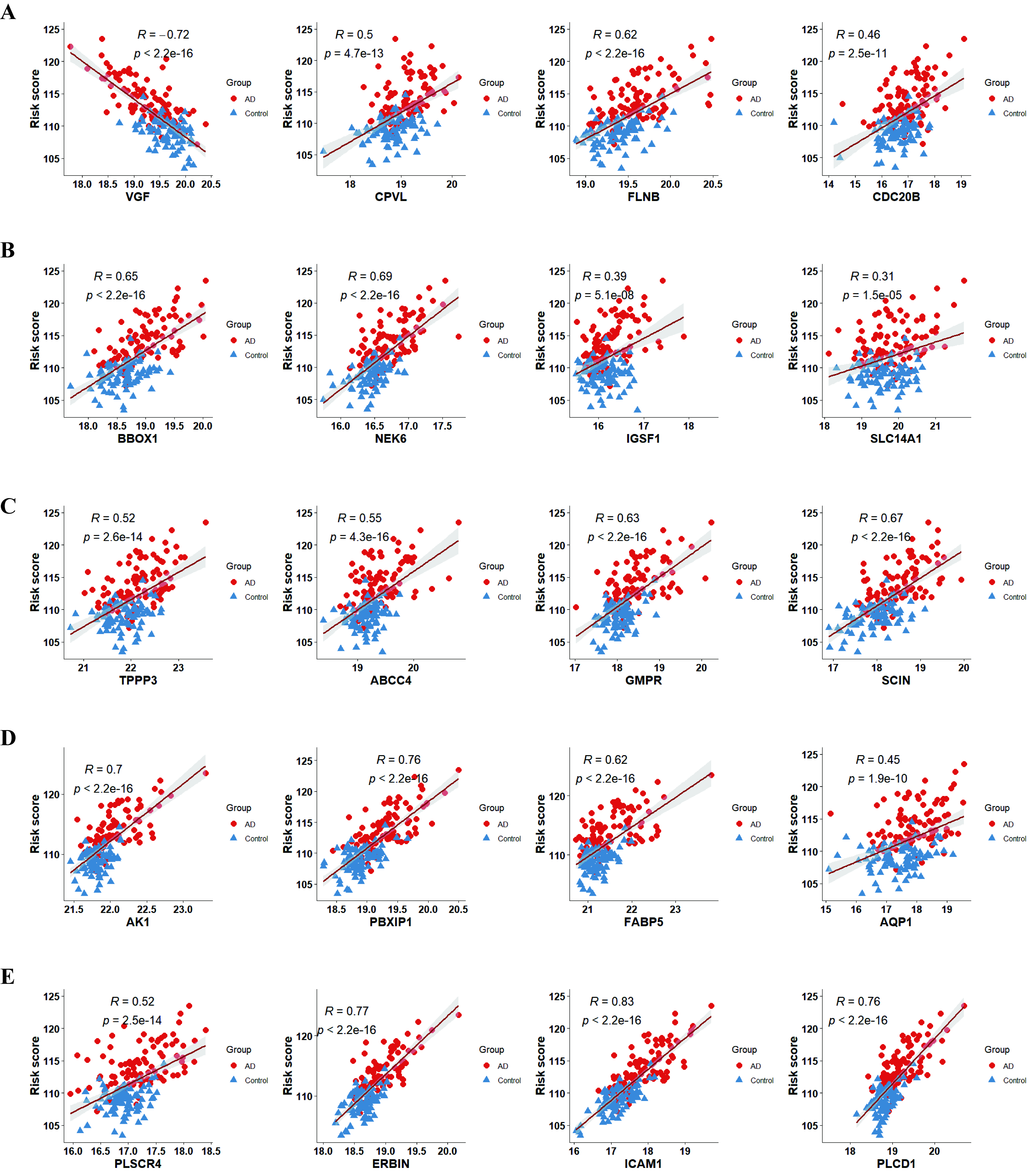

Supplement: Supplementary file 5 — Figure S5: Linear regression analysis of 20 hub proteins and risk scores. [file CNS-32-e71021-s001.tif]

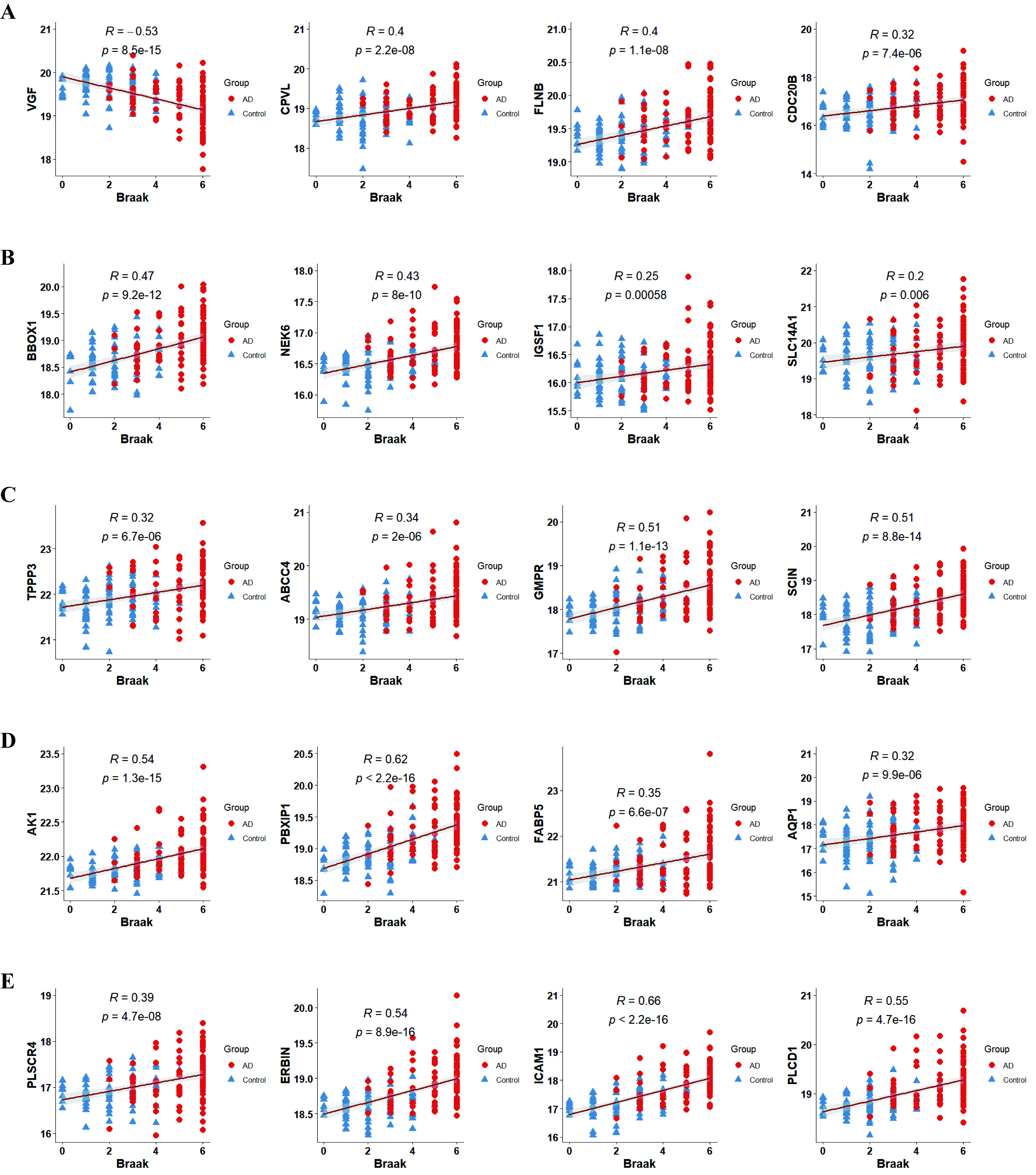

Supplement: Supplementary file 6 — Figure S6: The linear regression analysis of 20 hub proteins and Braak scores. [file CNS-32-e71021-s003.tif]

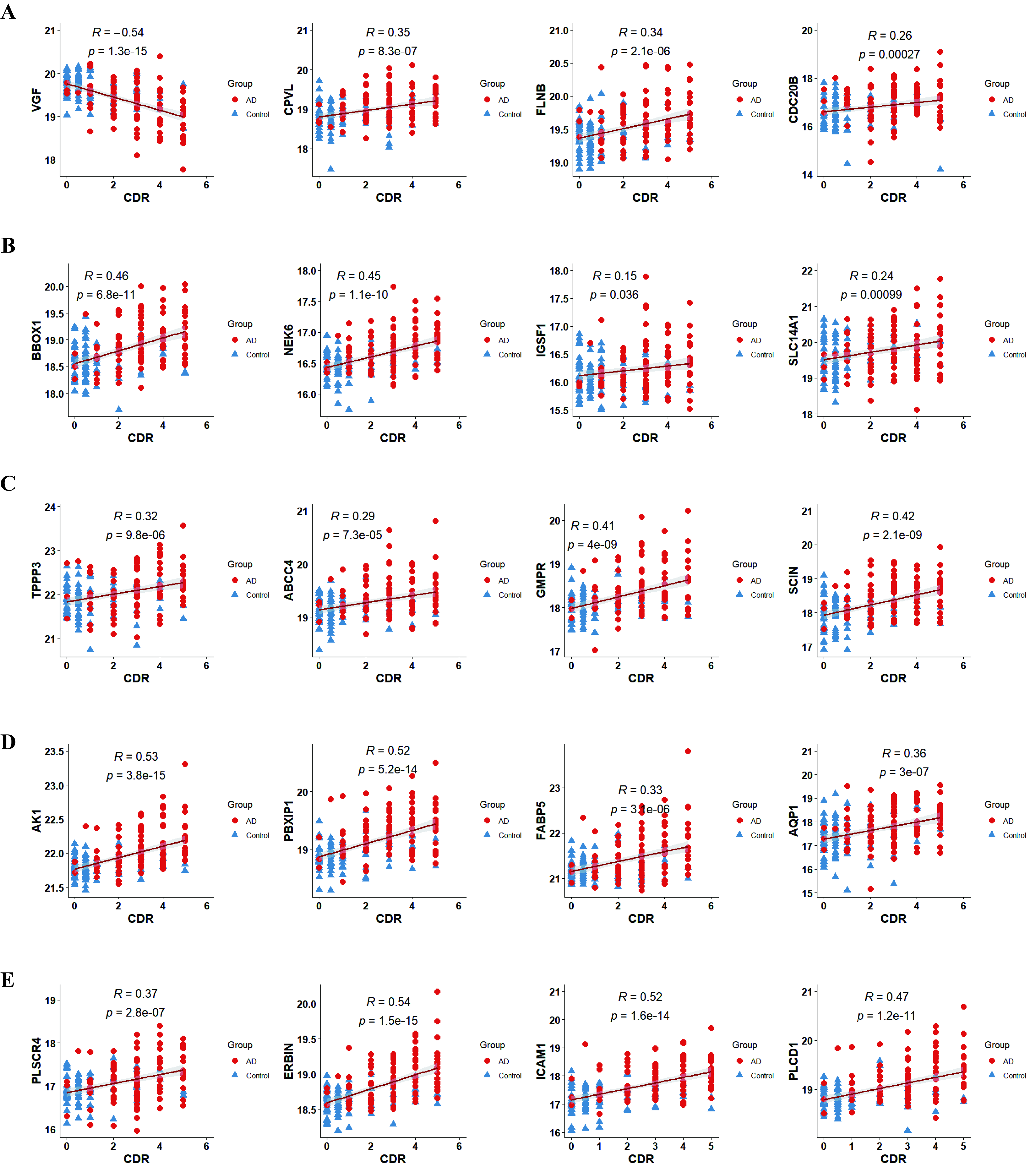

Supplement: Supplementary file 7 — Figure S7: The linear regression analysis of 20 hub proteins and CDR scores. [file CNS-32-e71021-s015.tif]

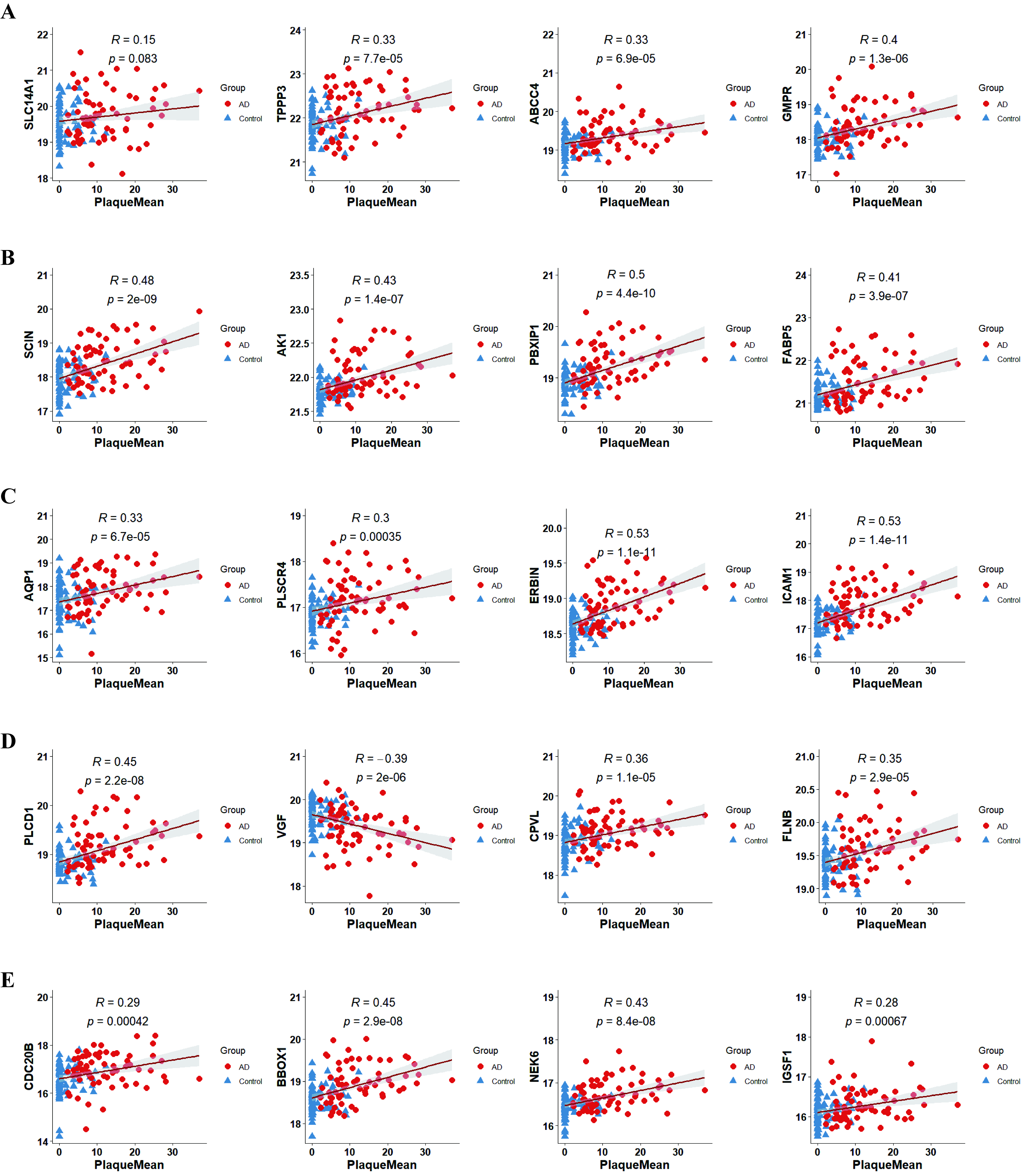

Supplement: Supplementary file 8 — Figure S8: The linear regression analysis of 20 hub proteins and PlaqueMean scores. [file CNS-32-e71021-s005.tif]

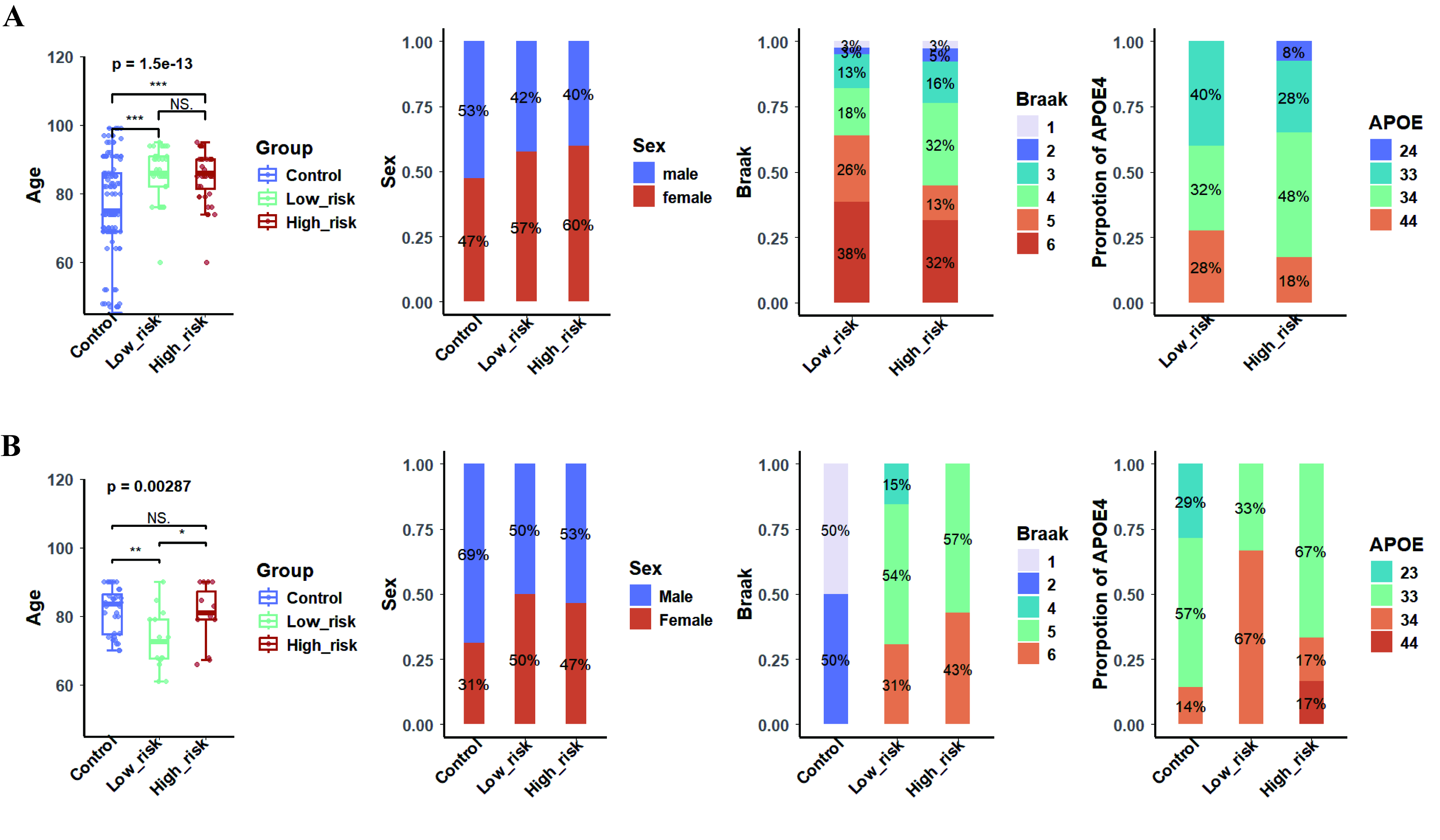

Supplement: Supplementary file 9 — Figure S9: Correlation analysis of clinical indicators in high‐ and low‐risk groups from external datasets. (A) Proportion of population distribution in control, high‐ and low‐risk groups, including age, gender, Braak, and APOE genotypes in GSE48350. (B) Proportion of population distribution in control, high‐ and low‐risk groups, including age, gender, Braak, and APOE genotypes in GSE29378. [file CNS-32-e71021-s009.tif]

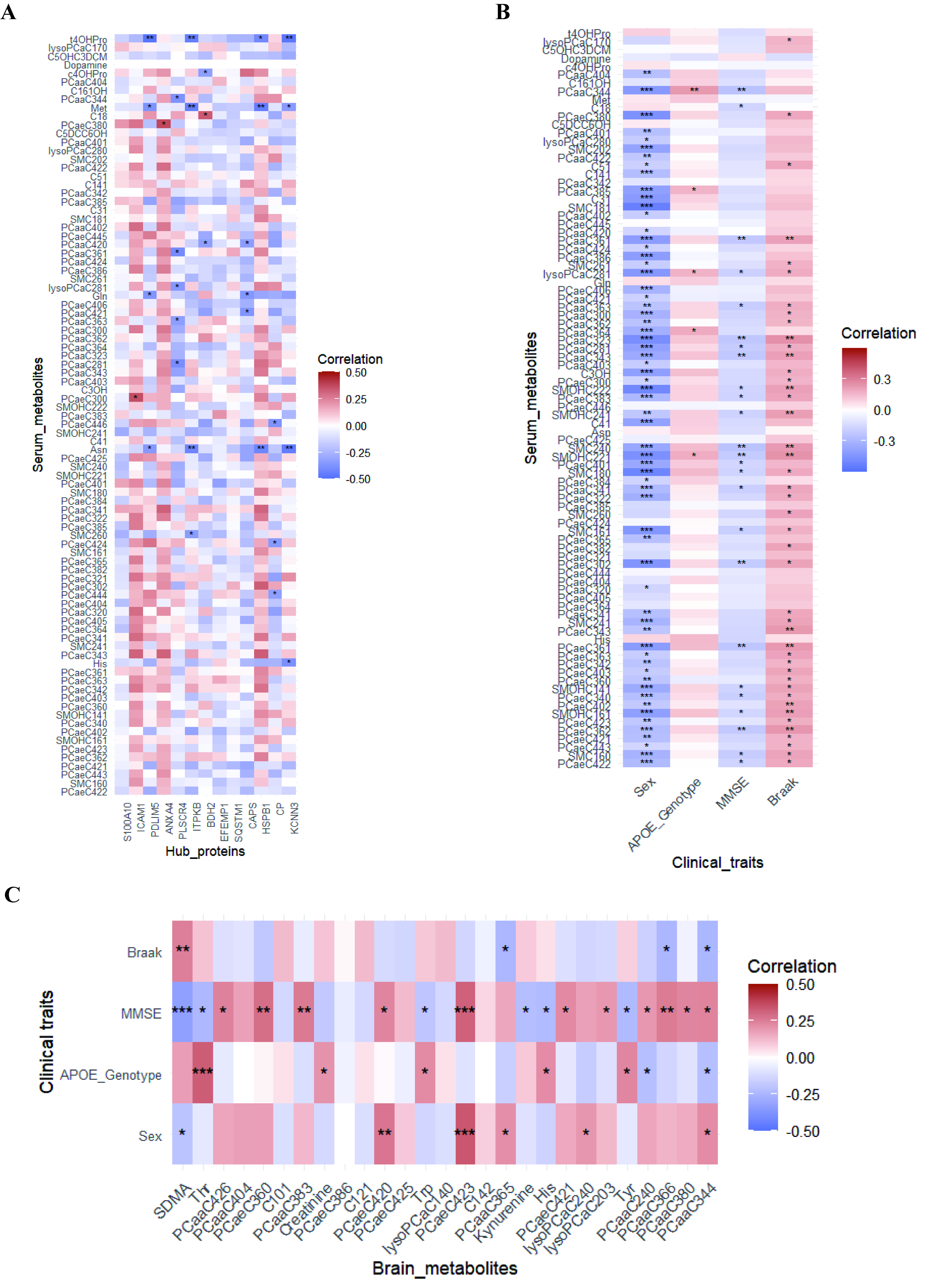

Supplement: Supplementary file 10 — Figure S10: Correlation analysis of differentially expressed metabolites in brain and serum. (A, B) Correlation heatmap of serum differential metabolites, hub proteins, and clinical indicators, p value: * < 0.05; ** < 0.01; *** < 0.001; **** < 0.0001. (C) Correlation heatmap showing the correlation analysis between differentially expressed metabolites in the brain and clinical manifestations of the disease (sex, APOE genotype, MMSE, Braak). p value: * < 0.05; ** < 0.01; *** < 0.001; **** < 0.0001. [file CNS-32-e71021-s008.tif]
